# Supplementary material for: Spatial heterogeneity of bone marrow endothelial cells unveils a distinct subtype in the epiphysis
Source: Nat Cell Biol. 2023 Oct 5;25(10):1415–25. doi: 10.1038/s41556-023-01240-7 (PMC10567563; doi:10.1038/s41556-023-01240-7)
Supplement: Supplementary file 1 — Gating strategy for Figs. 1i, 6d and 7a,h. [file 41556_2023_1240_MOESM1_ESM.pdf]

nature cell biology

Article https://doi.org/10.1038/s41556-023-01240-7

# Spatial heterogeneity of bone marrow endothelial cells unveils a distinct subtype in the epiphysis

In the format provided by the authors and unedited

## Fig. 1i

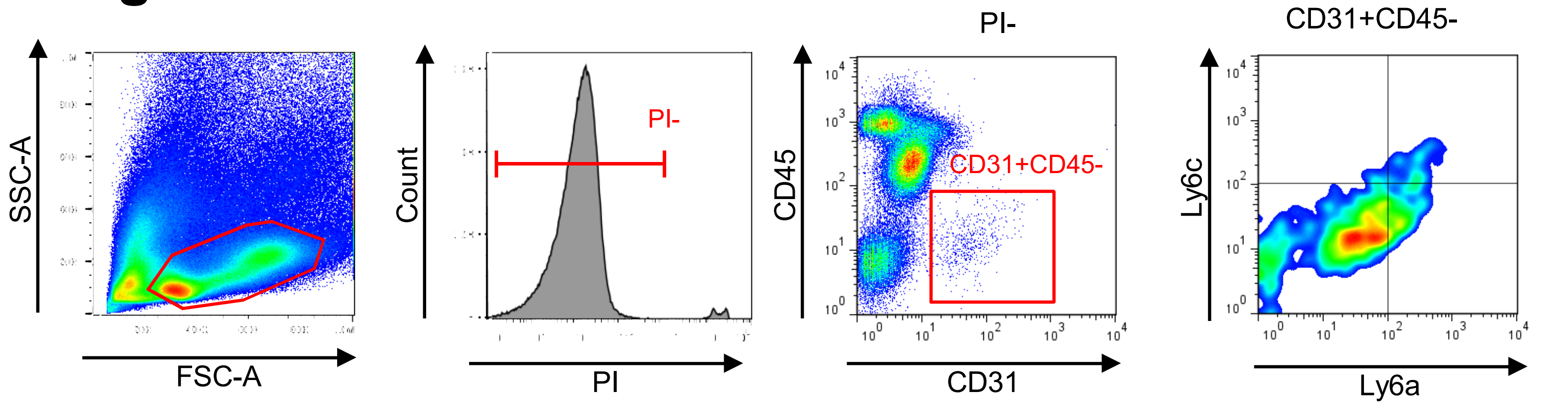


## Fig. 6d

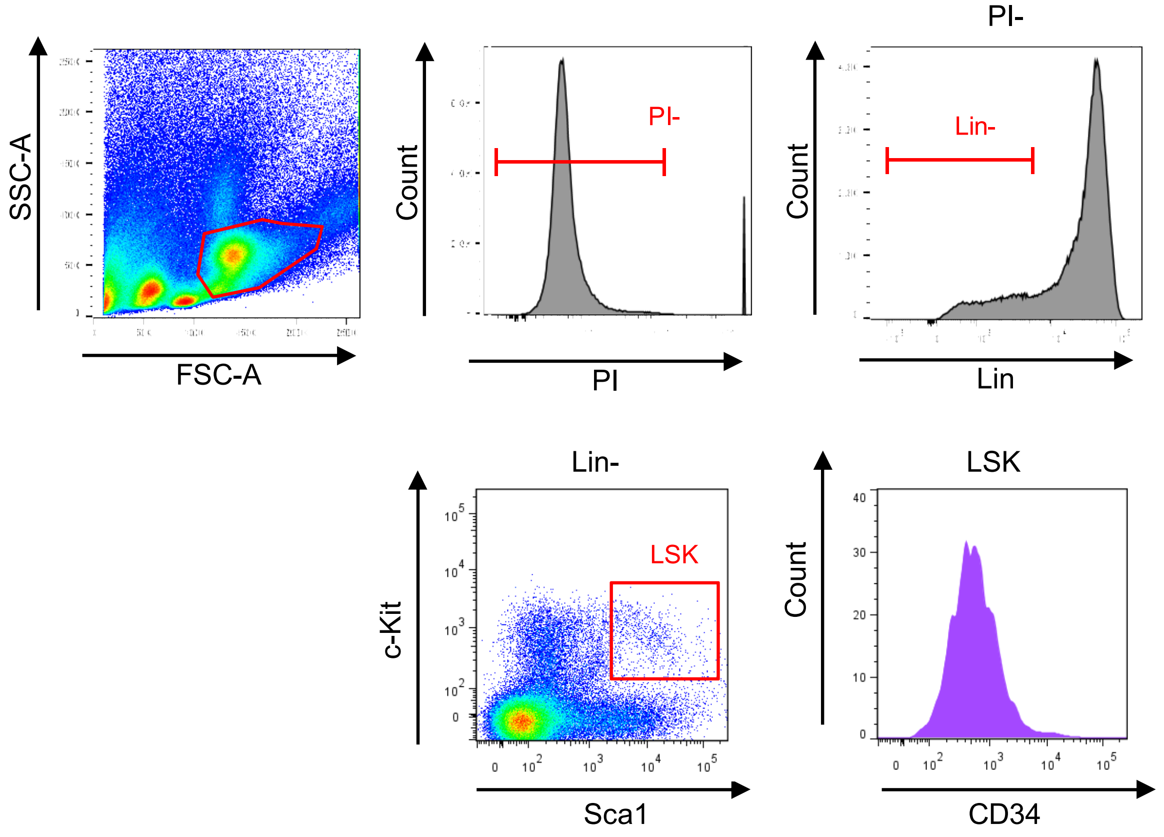


# Supplementary file: Gating strategy 1

# Fig. 7a

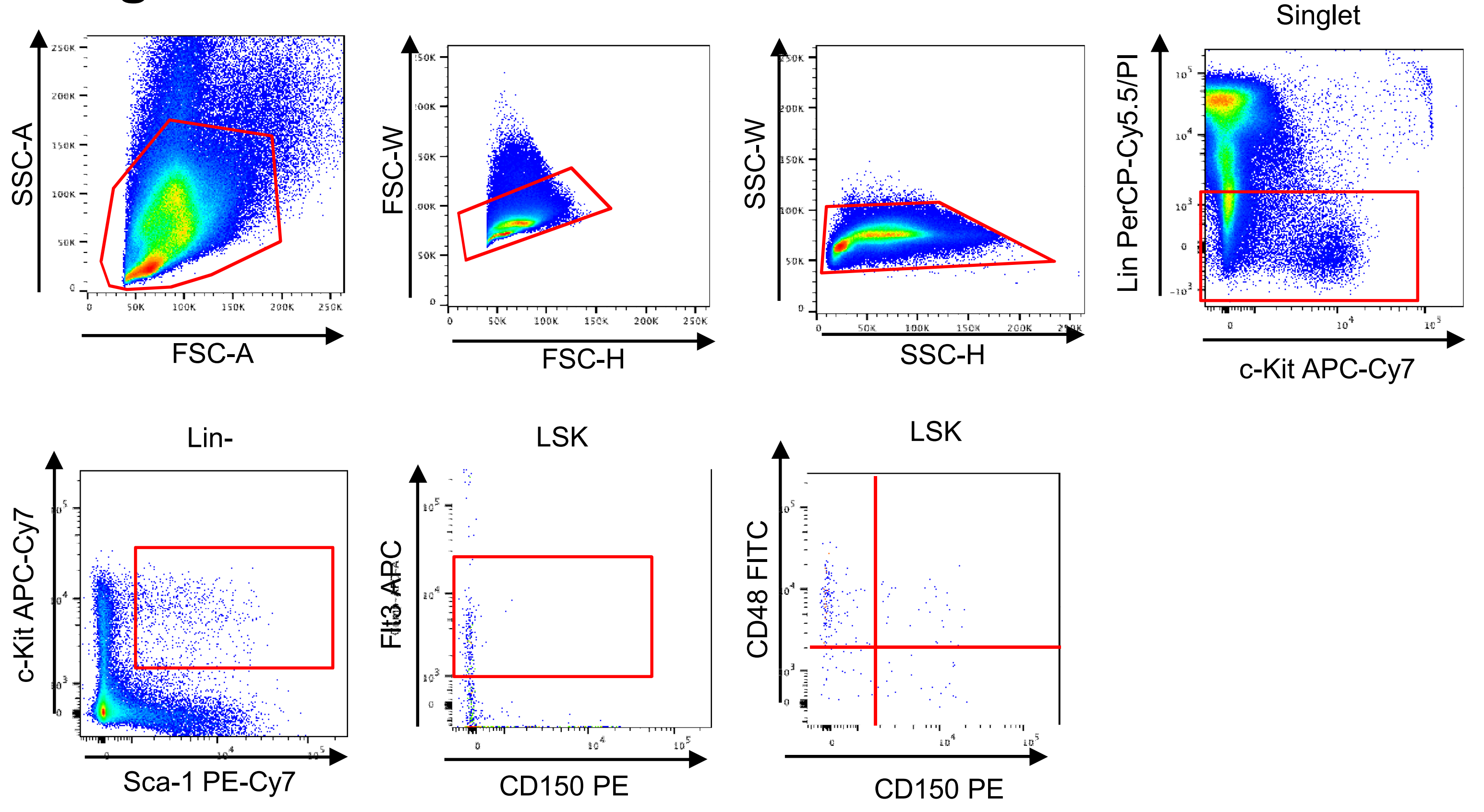


# Fig. 7h

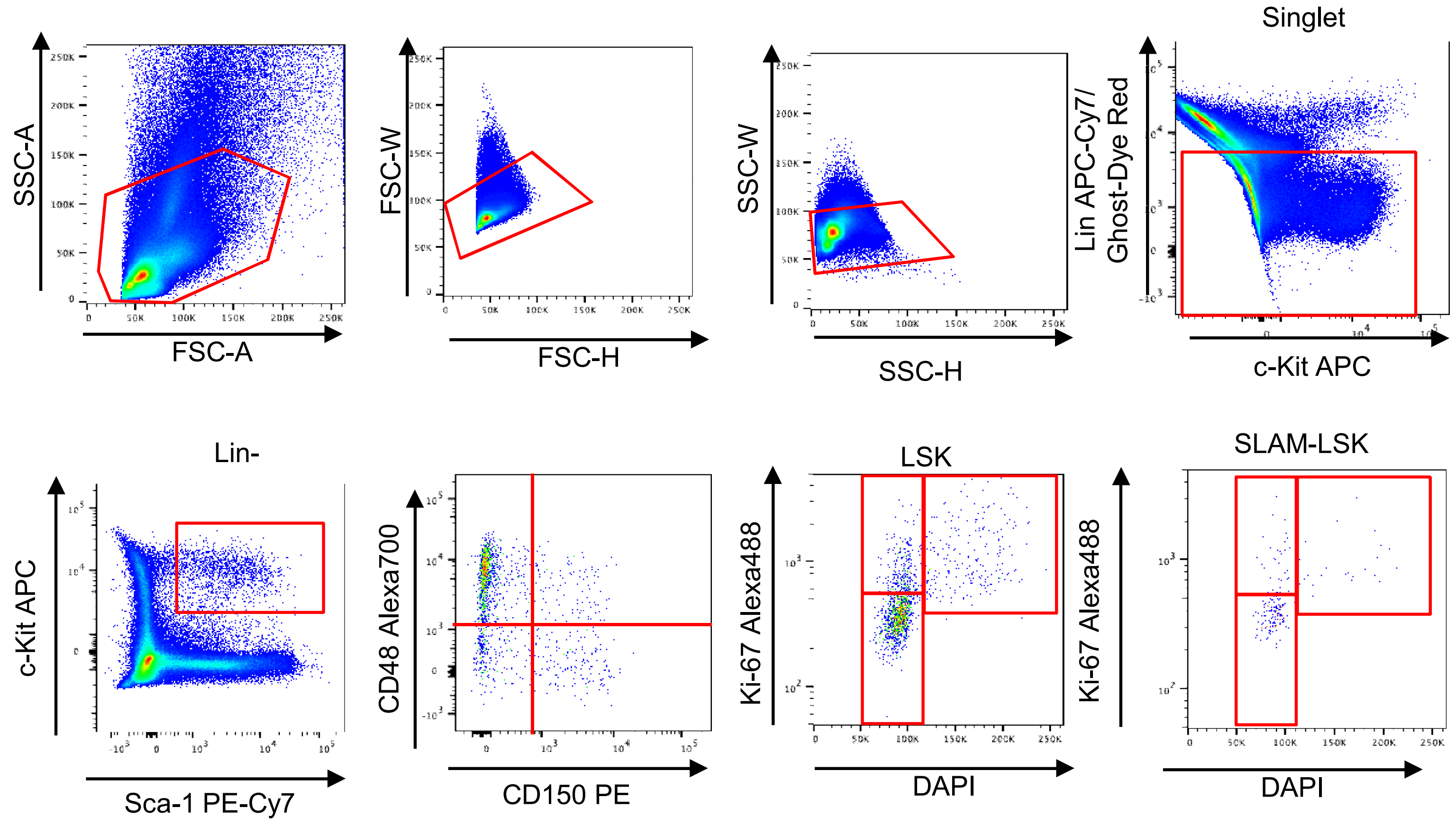


# Supplementary file: Gating strategy 2
