# Supplementary material for: Spatial heterogeneity of bone marrow endothelial cells unveils a distinct subtype in the epiphysis
Source: Nat Cell Biol. 2023 Oct 5;25(10):1415–25. doi: 10.1038/s41556-023-01240-7 (PMC10567563; doi:10.1038/s41556-023-01240-7)
Supplement: Supplementary file 2 — Reporting Summary [file 41556_2023_1240_MOESM2_ESM.pdf]

nature portfolio

Corresponding author(s): Yoshiaki Kubota

Last updated by author(s): Jul 14, 2023

Reporting Summary

Nature Portfolio wishes to improve the reproducibility of the work that we publish. This form provides structure for consistency and transparency in reporting. For further information on Nature Portfolio policies, see our Editorial Policies and the Editorial Policy Checklist.

Statistics

For all statistical analyses, confirm that the following items are present in the figure legend, table legend, main text, or Methods section.

n/a	Confirmed	
☐	☒	The exact sample size (n) for each experimental group/condition, given as a discrete number and unit of measurement
☐	☒	A statement on whether measurements were taken from distinct samples or whether the same sample was measured repeatedly
☐	☒	The statistical test(s) used AND whether they are one- or two-sided<br>*Only common tests should be described solely by name; describe more complex techniques in the Methods section.*
☒	☐	A description of all covariates tested
☒	☐	A description of any assumptions or corrections, such as tests of normality and adjustment for multiple comparisons
☐	☒	A full description of the statistical parameters including central tendency (e.g. means) or other basic estimates (e.g. regression coefficient) AND variation (e.g. standard deviation) or associated estimates of uncertainty (e.g. confidence intervals)
☐	☒	For null hypothesis testing, the test statistic (e.g. F, t, r) with confidence intervals, effect sizes, degrees of freedom and P value noted<br>*Give P values as exact values whenever suitable.*
☒	☐	For Bayesian analysis, information on the choice of priors and Markov chain Monte Carlo settings
☒	☐	For hierarchical and complex designs, identification of the appropriate level for tests and full reporting of outcomes
☒	☐	Estimates of effect sizes (e.g. Cohen's d, Pearson's r), indicating how they were calculated

Our web collection on statistics for biologists contains articles on many of the points above.

Software and code

Policy information about availability of computer code

Data collection	FV10-ASW 3.0 Viewer (Olympus),Strand NGS software v3.3 (Strand Life Sciences), Cell Ranger 3.0 software (10×Genomics)<br>FACS Aria II and FACS Aria IIIu instrument (BD Biosciences).
Data analysis	FV10-ASW 3.0 Viewer (Olympus), Image J Software 1.46 (NIH), Seurat package v3.5.2 (Seurat), FlowJo TM v10.8.1(BD Bioscience, elda package of R software ver 1.5.0 (R-project.org), MACSquant ver 2.1.1 (Miltenyi)

For manuscripts utilizing custom algorithms or software that are central to the research but not yet described in published literature, software must be made available to editors and reviewers. We strongly encourage code deposition in a community repository (e.g. GitHub). See the Nature Portfolio guidelines for submitting code & software for further information.

Data

Policy information about availability of data

All manuscripts must include a data availability statement. This statement should provide the following information, where applicable:

- Accession codes, unique identifiers, or web links for publicly available datasets
- A description of any restrictions on data availability
- For clinical datasets or third party data, please ensure that the statement adheres to our policy

For scRNAseq data, raw data is available in GEO (GSE163451). There is no restriction on data availability. Mus musculus (house mouse) genome assembly GRCm38

(mm10) from Genome Reference Consortium Numerical is available in https://www.ncbi.nlm.nih.gov/datasets/genome/GCF_000001635.20/. Numerical source data are provided with this study. All the other data supporting the findings of this study are available from the corresponding author on reasonable request.

Research involving human participants, their data, or biological material

Policy information about studies with human participants or human data. See also policy information about sex, gender (identity/presentation), and sexual orientation and race, ethnicity and racism.

Reporting on sex and gender	N/A
Reporting on race, ethnicity, or other socially relevant groupings	N/A
Population characteristics	N/A
Recruitment	N/A
Ethics oversight	N/A

Note that full information on the approval of the study protocol must also be provided in the manuscript.

Field-specific reporting

Please select the one below that is the best fit for your research. If you are not sure, read the appropriate sections before making your selection.

☒ Life sciences ☐ Behavioural & social sciences ☐ Ecological, evolutionary & environmental sciences

For a reference copy of the document with all sections, see nature.com/documents/nr-reporting-summary-flat.pdf

Life sciences study design

All studies must disclose on these points even when the disclosure is negative.

Sample size	No statistical method was used to calculate sample size. Sample size was determined to be adequate based on the magnitude and consistency of measurable differences between groups. For histological analyses in Figs.1,2, 3, 4, 5,6, and Extended Data Figure 1, 2, 3,4,5,6,7 at least three, but typically more, independent samples, were quantified or qualitatively analysed with each experimental repeat yielding highly similar results. Experimental animals were always compared to litter mate controls. For single cell RNA-seq (Fig. 1,4,5, and Extended Data Figure 1), the sample sizes were the maximum number of cells we could isolate from 20 mice. For Flow cytometry analysis (Fig. 1i), the sample sizes were the maximum number of cells we could isolate from 10 mice . For Flow cytometry analysis (Fig. 7), the sample sizes were the maximum number of cells we could isolate from 6 mice . The number of cells analyzed were sufficient because their numbers were high and isolated from all bones. No statistical methods were used to pre-determine sample sizes but our sample sizes are similar to those reported in previous publications (Okabe et al., 2014; Tai-Nagara et al., 2020). Data distribution was assumed to be normal but this was not formally tested.
Data exclusions	No data was excluded.
Replication	We have reproduced all our results in a minimum of three independent experimental repeats and using independent samples. Many experiments were repeated independently by two investigators in the lab, in particular experiments shown in Fig. 1, 2, 3, 4, 5, 6,7, and Extended Data Figure 1, 2, 3, 4,5,6. All attempts at replication were successful.
Randomization	Randomization was not required for our mouse studies because we determined the genotype using genomic PCR before the experiments.
Blinding	PCR genotyping determined group allocation, and the investigators were not blinded in this process. Data analysis was performed by different lab members involved in each analysis, to avoid conscious and unconscious bias.

Reporting for specific materials, systems and methods

We require information from authors about some types of materials, experimental systems and methods used in many studies. Here, indicate whether each material, system or method listed is relevant to your study. If you are not sure if a list item applies to your research, read the appropriate section before selecting a response.

Materials & experimental systems

n/a	Involved in the study
☐	☒ Antibodies
☒	☐ Eukaryotic cell lines
☒	☐ Palaeontology and archaeology
☐	☒ Animals and other organisms
☒	☐ Clinical data
☒	☐ Dual use research of concern
☒	☐ Plants

Methods

n/a	Involved in the study
☒	☐ ChIP-seq
☐	☒ Flow cytometry
☒	☐ MRI-based neuroimaging

Antibodies

Antibodies used

The primary monoclonal antibodies used were, CD31 (Abcam; Cambridge, UK, ab119341; 1:1000), Runx2 (Abcam; ab192256; 1:500), ASMA (Sigma–Aldrich, Saint Louis, MO, USA, A5228; 1:200), Ly6a (BD; 553333; 1:500), anti-CD150 (BioLegend, San Diego, CA, USA; 115905; 1:200), and Endomucin (Santa Cruz; Santa Cruz, CA, USA; sc-65495; 1:500). The primary polyclonal antibodies used were as follows: GFP-Alexa Fluor 488-conjugated (Molecular Probes, Eugene, OR, USA, A21311; 1:500), c-Kit (R&D Systems, Minneapolis, MN, USA, AF1356; 1:500), Sp7/Osterix (Abcam; ab22552; 1:500), Cathepsin K (Abcam; ab19027; 1:500), VEGFR3 (R&D; AF743; 1:1000), CGRP (SIGMA; C8198; 1:8000), and anti-Erg (Abcam, ab92513, 1:2,000). Secondary antibodies used were Alexa Fluor 488-conjugated IgGs (Molecular Probes, A11034, A11006, A11055; 1:500) or Cy3/Cy5 DyLight549/DyeLight649-conjugated IgGs (Jackson ImmunoResearch, West Grove, PA, USA, 711-165-152, 112-165-167, 127-165-160, 711-605-152, 112-605-167, 127-605-160; 1:500). For nuclear staining, specimens were treated with 4',6-diamidino-2-phenylindole (DAPI; Molecular Probes, D-1306). For Flow cytometry analysis, we used the following antibodies. FITC-conjugated anti-CD31 antibodies (Biolegend; 102506; 1:200), APC-conjugated anti-CD45 antibodies (Biolegend; 103112; 1:200), PE-conjugated anti-Ly6a antibodies(Biolegend; 122507; 1:200), and APC/Cy7-800 conjugated anti Ly6c antibodies(Biolegend; 128025; 1:200)

Validation

All antibodies were sold by the manufacturer with validation data and citations, and they detected the specified targets in our study as expected. Specificity was confirmed by obtaining the expected pattern of tissue staining with the respective antibodies.

All commercially available antibodies are routinely tested by he manufacturers.
-CD31 (Abcam; Cambridge, UK, ab119341; 1:1000). It has been successfully used for IF in the mouse mesentery by Deng Muller's group (doi: 10.1084/jem.179.3.1059).
-Runx2 (Abcam; ab192256; 1:500). It has been successfully used for IF in the mouse dura by Elizabeth Engles's group (doi: 10.1016/j.devcel.2017.07.027.).
-ASMA (Sigma–Aldrich, Saint Louis, MO, USA, A5228; 1:200). It has been successfully used for IF in the mouse carotid artery by Adria Giacca's group (doi: 10.1161/ATVBAHA.109.185447).
-Ly6a (BD; 553333; 1:500). It has been successfully used for IF in the mouse bone marrow by Ralf Adams's group (doi:10.1038/nature17638).
-anti-CD150 (BioLegend, San Diego, CA, USA; 115905; 1:200). It has been successfully used for IF in the mouse bone marrow by Paul Frenette's group (doi: 10.1038/nature12612.).
-Endomucin (Santa Cruz; Santa Cruz, CA, USA; sc-65495; 1:500). It has been successfully used for IF in the mouse bone marrow by Ralf Adams's group (doi:10.1038/nature17638).
-GFP-Alexa Fluor 488-conjugated (Molecular Probes, Eugene, OR, USA, A21311; 1:500). It has been successfully used for IF in the mouse bone marrow by Ralf Adams's group (doi:10.1038/nature17638).
-c-Kit (R&D Systems, Minneapolis, MN, USA, AF1356; 1:500). It has been successfully used for IF in the mouse bone marrow by Paul Frenette's group (doi: 10.1038/nature12612.).
-Sp7/Osterix (Abcam; ab22552; 1:500). It has been successfully used for IF in the mouse bone marrow by Ralf Adams's group (doi:10.1038/nature13145).
-Cathepsin K (Abcam; ab19027; 1:500). It has been successfully used for IF in the mouse digits by Ken Muneoka's group (doi: 10.1016/j.ydbio.2022.03.007).
-VEGFR3 (R&D; AF743; 1:1000), CGRP (SIGMA; C8198; 1:8000). It has been successfully used for IF in the mouse bone marrow by Ralf Adams's group (doi:10.1038/nature13146).
-anti-Erg (Abcam, ab92513, 1:2,000). It has been successfully used for IF in the mouse lung by Ralf Adams's group (doi:10.1038/s41467-017-01738-3).
-FITC-conjugated anti-CD31 antibodies (Biolegend; 102506; 1:200). It has been successfully used for flowcytometry in the mouse bone marrow by Ralf Adams's group (doi:10.1038/nature17638).
-APC-conjugated anti-CD45 antibodies (Biolegend; 103112; 1:200). It has been successfully used for flowcytometry in the mouse bone marrow by Ralf Adams's group (doi:10.1038/nature17638).
-PE-conjugated anti-Ly6a antibodies (Biolegend; 122507; 1:200). It has been successfully used for flowcytometry in the mouse bone marrow by Rubinsztein DC's group (10.1038/ncomms10533).
-APC/Cy7-800 conjugated anti Ly6c antibodies (Biolegend; 128025; 1:200). It has been successfully used for flowcytometry in the mouse bone marrow by Rubinsztein DC's group (10.1038/ncomms10533).

Animals and other research organisms

Policy information about studies involving animals; ARRIVE guidelines recommended for reporting animal research, and Sex and Gender in Research

Laboratory animals	The strains (Cdh5-BAC-CreERT2, Osx-CreERT2,FVegfr2-flox, Dll4-flox, Col1a1-flox,CAG- LSL-EGFP, Vegfr1-BAC-DsRed ,Vegfr2-BAC-GFP, Rbpj-flox, Csf1op/op,Cxcl12+/DsRed) have all been published previously and are described in the methods section with appropriate references. Mice at P7, P11, P13, P18, P28, P35, 3Mo, 4Mo, and 6Mo. Ages of mice used in each experiment are specified in the figures and figure legends. Mouse cages were well ventilated, softly lit and subject to a 12h light/dark cycle. The relative humidity were kept at 45 to 65%. Mouse rooms and cages were kept at a temperature range of 20-24oC.
Wild animals	The study did not involve wild animals.
Reporting on sex	The vast majority of work was carried out with neonatal materials of unknown sex, but, based on Mendelian genetics, all material would be comprised of equal proportions of male and female tissues, as none of the strains we have used have reported sex-related lethality that would eliminate one or the other sex from litters. The sex of the tissues has been recorded and can be provided if required, but is considered not relevant in the context of our study.
Field-collected samples	The study did not involve samples collected from the field.
Ethics oversight	Animal experiments were approved by the Institutional Animal Care and Use Committee of Keio University (protocol number A2021-003) and were performed in accordance with the Guidelines of Keio University for Animal and Recombinant DNA Experiments (protocol number D2005-044).

Note that full information on the approval of the study protocol must also be provided in the manuscript.

Flow Cytometry

Plots

Confirm that:

- [x] The axis labels state the marker and fluorochrome used (e.g. CD4-FITC).
- [ ] The axis scales are clearly visible. Include numbers along axes only for bottom left plot of group (a 'group' is an analysis of identical markers).
- [x] All plots are contour plots with outliers or pseudocolor plots.
- [x] A numerical value for number of cells or percentage (with statistics) is provided.

Methodology

Sample preparation	Femurs from wild-type mice at postnatal 12 weeks were collected in PBS+2%FCS.Epiphysis, and metaphysis were crushed separately, and diaphysis was flushed to collect bone marrow cells
Instrument	Cell sorting was performed using a FACS Aria II, FACS Aria IIIu instrument (BD Biosciences).or SH800S (SONY)
Software	Data were analyzed with FlowJo software (Treestar)
Cell population abundance	Post-sorted cells were immediately re-analyzed to confirm the validity and purity (>95%) of each sorting.
Gating strategy	Debris and dead cells were excluded by forward and side scatter and a negative gate for propidium iodide staining. Percentages of cell doublets/aggregates were checked by FSC-A vs. FSC-W, and SSC-A vs. SSC-W plots. In all FACS studies, we routinely included unstained control samples and compensation tubes. Using these negative and positive control tubes, we set fluorescence voltages and the compensation matrix according to the instructions of the manufacturer.

- [x] Tick this box to confirm that a figure exemplifying the gating strategy is provided in the Supplementary Information.
